# Spelling across Tasks and Levels of Language in a Transparent Orthography

**DOI:** 10.1371/journal.pone.0163033

**Published:** 2016-09-22

**Authors:** Lucia Bigozzi, Christian Tarchi, Giuliana Pinto

**Affiliations:** Department of Education and Psychology, University of Florence, Florence, Italy; Kyoto University, JAPAN

## Abstract

The paper reports the results of two studies on the spelling performance of 1^st^ graders in a transparent writing system. The spelling performance of Italian children was assessed to determine the cross-task relationship between spelling to dictation and spontaneous spelling at the single word level (Study 1) and at the text level (Study 2), respectively. In study 1, 132 Italian children’s spelling performance was assessed in 1^st^ grade through two standardized tasks, i.e., word dictation, and spontaneous word spelling. In study 2, spelling performance of 81 Italian children was assessed in 1^st^ grade through two tasks, i.e., text dictation, and spontaneous text spelling. In Study 1, spelling words and pseudo-words to dictation was found to be more difficult than spontaneous spelling of words. This effect was verified for all children (including low achievers and spelling impaired). The moderate correlation found between spelling to dictation and spontaneous spelling indicated that the two tasks are supported by partially different spelling processes and confirmed suggestions for including both types of spelling assessments in the school. In Study 2, children's spelling performances were not dependent across the two tasks (i.e., spelling a text under dictation or spontaneously). The two tasks shared the level of difficulty but performance in one task was not predictive of performance in the second task. Strong individual differences between children were found at the text level as a function of task. Similar to Study 1, the moderate correlation between spelling text to dictation and spontaneous spelling confirmed the usefulness of adopting both spelling assessments at school.

## Introduction

Spelling plays a fundamental role in the writing process, especially in early stages of formal literacy acquisition [1]. Spelling is a complex cognitive activity, involving the integration of motor, linguistic, and memory processes [2]. Because many young students experience difficulties in the spelling phase, which in turn limits the overall writing process [3,4], it is crucial to assess spelling performance as early as possible, in order to detect difficulties that might impair children's writing skills. Indeed, analyzing whether spelling performance varies by task (e.g., dictation or spontaneous spelling) and level of language (e.g., word and text) could inform us on the nature of spelling in its early stage of acquisition [2,5], and help interpret spelling problems [2,6,7]. However, research analyzing children's spelling performance is scarce [2], and even less research is available comparing different spelling assessment methods [8]. Literature on text writing has mainly focused on composition, rather than spelling or on text dictation. Scholars have either analyzed spelling at the word level, or when focusing on the text level, they have privileged research on the content, partially neglecting the analysis of spelling.

This research verifies whether spelling accuracy during the initial stages of spelling acquisition (*i*.*e*., 1^st^ grade) within an orthographically shallow language (i.e., Italian) is consistent across assessment tasks (dictation vs. spontaneous spelling) and language levels (word vs. text). This study contributes to our understanding of children’s early acquisition of spelling. Teachers assess children’s spelling performances with a variety of tasks which differ for important characteristics. The act of spelling a spoken word to dictation requires the student to integrate central spelling processes (*i*.*e*., retrieving, assembling and selecting an orthographic representation), and peripheral spelling processes (i.e., producing an output and executing the orthographic codes) [9]. Instead, the act of spontaneously spelling a word requires the student to integrate transcoding skills with text generation skills (i.e., idea generation and translation of ideas into language representations in working memory) [10]. Studying early spelling acquisition is particularly important because it is considered to be a reflection of orthographic learning [11].

### Spelling skills in primary school

Spelling is the process of transcoding sounds (or mental representations of sounds) to signs. According to the dual route model [12–15], a theory based on English learners, there are two spelling procedures available: the lexical procedure, in which the speller has access to word-specific orthographic representations [16], and the sub-lexical procedure, in which the speller relies on the rule-driven conversion of phonemes into graphemes [17]. Originally, this model was proposed by Coltheart, Patterson and Marshall [18] for skilled adult reading, but was then adapted to spelling by Patterson [19]. Firstly, the speller needs to analyze the auditory stimulus. Then the expert speller activates the lexical route to retrieve the orthographic representation of the word from the semantic storage, and sends it to short-term storage defined as a graphemic buffer. The sub-lexical route converts the string of phonemes into a string of graphemes and then sends it to the graphemic buffer. This strategy is used by the speller for new words. The orthographic representation is stored in the graphemic buffer for all the time needed to write its motor realization [20]. In a developmental perspective, in early stages of spelling acquisition, children rely on the sub-lexical route, and only subsequently do they learn to spell through the lexical one [21]. Children tend to spell through the sub-lexical route for several years, and rely on the lexical one only once they have stored a relatively great amount of orthographic representations [22].

### Spelling performance as a function of task

Spelling is a process often assessed and graded in school, with several different measures and tasks, for instance dictation (*i*.*e*., the act or manner of transcribing words uttered by another) or spontaneous spelling. Few studies have explored children’s spelling performance as a function of task. Molfese, Beswick, Molnar, and Jacobi-Vessels [23] compared spelling-to-dictation versus spelling-to-copying in 79 English-speaking preschool children, and found that children obtained higher scores in the copying task than they did in the dictation task. Puranik and Apel [2] compared 104 English-speaking preschoolers’ spelling performances in three tasks (i.e., written word spelling, oral word spelling, and tile spelling task), and found that spelling performance did not vary by task if children had already achieved a good mastery of spelling skills.

To the best of our knowledge no prior research has focused specifically on early spelling skills as a function of task, or compared spelling-to-dictation with spontaneous spelling. However, it is an important gap to fill because, although several types of measures might be associated with spelling skills, some measures might capture more variance than others [8]. Previous studies have demonstrated that at the text-level composition and spelling processes influence each other [3]. This study could indicate a difference in assessing spelling performance, which would inform practitioners on the different degrees of difficulties of this task, or alternatively spelling performances might be similar across tasks, which would suggest to practitioners the possibility of reflecting on when one task might be more appropriate than another. For instance, dictation might be better suited to assess the use of lexical and sub-lexical processing, whereas composition might be better suited to assess whether the construction of a narrative interferes with spelling performances. Past studies [24–26] demonstrated that spelling competence in first grade disrupts the continuity from oral narrative competence in kindergarten to written narrative competence in first and second grade. Thus, in this study we also assessed the quality of students' narratives at the macro-level, i.e., the overall interrelatedness in the text (coherence, [27]), and the activation of conventional narrative components (structure, [28,29]).

### Spelling performance as a function of levels-of-language

Recent studies have analyzed spelling as a function of levels-of-language, and successfully demonstrated that children’s learning processes work differently when implemented at the word and text level [30–32]. With this approach, spelling performance might vary depending on whether one is asked to spell words or write texts. Listing words activates the lexical route, whereas listing pseudo-words activates the sub-lexical one. Writing a text instead, requires one to be accurate in spelling while, at the same time, to generate ideas and words. Our understanding of how spelling performances in spelling changes as a function of task is limited by the fact that research on this topic was rather fragmented and included different components of writing (*i*.*e*., spelling and composition) or different spelling processes (*i*.*e*., in reading and writing). To the best of our knowledge no prior research has focused specifically on early spelling skills in writing as a function of levels of language.

Zoccolotti, Luca, and Spinelli [33] suggested that reading deficits might not be entirely explained at the word level: when processing texts, children need to integrate the processing of words with other processes of reading, which might additionally affect their cognitive abilities. Thus, we wanted to test this hypothesis in spelling too. Ahmed et al. [31] investigated the longitudinal relationships between reading and spelling at the word, sentence, and text levels for English-speaking 1^st^-4^th^ graders. The authors found that at the word and text levels, reading exerted a relatively larger influence on spelling than vice versa, but at the sentence level, reading, and spelling appeared to reciprocally influence each other. The authors concluded that children apply the knowledge acquired through the reading process to their spelling across all levels of language, whereas the reverse process (spelling influencing reading) is not likely to occur. Finally, one study explored spelling disabilities as a function of levels of language. Mayes et al. [7] analyzed 54 English-speaking children, who had been referred to the school psychologist. Transcription (i.e., word dictation, and sentence construction) and composition (i.e., compose a letter) were used to test children's spelling skills. They used the tests to classify children as having a spelling disability, if they were scoring significantly lower than predicted by their WISC-III IQ score. The results demonstrated that the percentage of spelling disabilities identified by the composition test was significantly higher (78%) than the percentage obtained by the dictation (28%) and sentence construction task (11%). They concluded that spelling assessments can influence the identification of a disability.

At the word level of language, spelling under dictation or spelling spontaneously should share the same underlying mental processes. In both tasks the orthographic representation of a word needs to be kept in the graphemic buffer and translated into a motor act. In the dictation task, words arrive from the graphemic buffer under someone else’s input, whereas in the spontaneous spelling task it is the speller him- or herself who sends the word to the graphemic buffer. The only difference lies in the auditory processing of the word that the spellers need to write in the dictation task.

At the text level instead, the spontaneous spelling task involves more processes that are not involved in the dictation task: planning, monitoring, and revising, involvement of long-term memory, attention, task environment, and motivational aspects [34]. Some children are extremely accurate in both tasks, because the spelling process has been automatized and does not require the implementation of cognitive resources, making them available for the composition process. Other children might be accurate at the word level of language, but might make mistakes at the text level of language. In this case, their orthographic accuracy at the word level might have been achieved through the implementation of several cognitive resources, which are no longer available when composing a text. Other children spell without making mistakes because when composing a text, they retrieve words from their semantic storage, whose orthographic representation is available through the lexical route. Instead, in a dictation task they cannot rely on such a strategy, and might struggle more and make more spelling mistakes.

### Spelling in shallow orthographies

The dual route model was originally developed for English-speaking learners, and it might not fully explain spelling acquisition in all orthographies. Alphabetic orthographies are on a continuum according to the transparency of their grapheme-phoneme correspondence. Shallow orthographies are characterized as having nearly a 1:1 correspondence, whereas in deep orthographies the grapheme-phoneme correspondence is equivocal [35]. The orthographic consistency of a language may influence the use of the lexical and sub-lexical routes as a function of literacy acquisition. Whereas the effect of orthographic consistency on reading acquisition is well documented in literature [36], cross-linguistic studies exploring the effect of orthographic consistency on spelling acquisition are more limited [15]. The few studies available support the dual route model for spelling acquisition in transparent orthographies too [5,37], although some differences exist. Spelling acquisition is faster in transparent orthographies than what happens in opaque orthographies [38–40]. Besides the ease of spelling acquisition, orthographic consistency influences the use of different spelling procedures too [41]. In Italian, a transparent orthography, despite some early evidence of use of the lexical procedure in the first grades [5,42], in early stages of spelling acquisition children mainly rely on the sub-lexical route, and become very accurate by the end of the first grade [5]. Overall, for transparent orthographies, data indicate an early and rapid development of the sub-lexical route, and a more gradual acquisition of the lexical route [15,41].

Overall, shallow orthographies provide low potential for spelling errors at the level of words, and higher potential for spelling errors at the level of text. For instance, in Italian, at the level of words when the phoneme [k] precedes the phoneme [w], it can have to different spellings: <c> or <q>, depending on the word: *cuore* ['kwɔre] (heart) and *quadro* ['kwadro] (painting) [43]. Or, for example, the syllables [t∫e], [∫e], [dʒe] may, or may not, require the grapheme <i>: [∫ena] is spelled as *SCENA* (= scene), but [∫entsa] is spelled as *SCIENZA* (= science) [5]. However, these represent regular rules to be learned. At the level of text, instead, the writer can possibly encounter spelling ambiguities that are context-sensitive. For instance, *anno* (year) or *hanno* (they have) are homophone but not homograph words, but only the context of the sentence in which they are included can help the writer to decide which spelling is the correct one. Even among shallow orthographies differences can exist. For instance, [44] reported only a very few words spelled incorrectly in the composition task by first graders, but attributed this result to the Finnish language's extremely regular orthography.

### Rationale and research questions

This research analyses Italian 1^st^ graders’ spelling performance in two different tasks and across two different language levels. First grade represents an important transition in the formal acquisition of written language, as the mechanics of spelling weigh more in early stages, when the process is still not automatic, and spelling difficulties are still not rooted. Two studies were designed, and consisted of two repeated cohorts. Cohort studies include all children from the natural population, at-risk and not for learning disorders. This approach allows a better control of potentially confounding variables (e.g., socio-economic status or teaching style) and increases the reliability of the studies. In particular, the two cohorts were extracted from the same school district. In both studies, spelling performance was measured as the number of orthographic errors committed in relation to the number of words written, as done in several prior studies on spelling in text composition (see for instance [45,46]). We kept the same measure across the four tasks (dictation of words and text, spontaneous spelling of words and text) to increase the comparability in performances, and also because we were not interested in understanding which task was more difficult (a result that could be biased by ratios), but rather in determining whether levels of inaccuracy depend on the task or not.

The study results will inform whether spelling competencies are consistent across tasks in different language levels: words (Study 1) and text (Study 2). In Study 1, children's spelling performance in a dictation test was compared to a spontaneous spelling test at the word level. In Study 2, children's spelling performance in a dictation test was compared to a spontaneous spelling test at the text level.

To further explore data, we also identified poor spellers (children with low achievement in spelling tests) and verified whether their spelling competence was consistent across tasks, and/or language levels, when compared to good spellers. However, defining what “low achievement” means in learning processes is a much debated issue. Policy-makers and scholars suggest the use of percentile, rather than standard deviation from the mean, but what should the threshold to identify low achievement in spelling be? The DMS-5 suggests the use of the 5^th^ percentile for the identification of learning disabilities [47]. In this study, besides children with a learning disability, we also wanted to include low achievers (children without a learning disability but nevertheless struggling in this process), so it was necessary to push this threshold forward. Several studies adopted the 25^th^ percentile as a threshold for low achievement in learning processes [48], but Wong and Butler [49] warned that such high thresholds might include students with unidentified intellectual disabilities or an unknown proportion of exclusionary criteria, and suggested the use of the 15^th^ percentile. In synthesis, our sample of poor spellers includes both low achievers (spelling performance between the 15^th^ and 5^th^ percentile) and children who might have a learning disability with a specific impairment in spelling (spelling performance lower than the 5^th^ percentile).

For Study 1 we predicted that spelling performance would be consistent across tasks. Indeed, studies on Italian spelling showed a presence for both the lexical and sub-lexical routes from early written language acquisition stages [5]. Typically, children rely on their lexical route for word dictation, and on the sub-lexical route to code unfamiliar words, for instance in a pseudo-word dictation task, whereas they use a mixture of these strategies in the spontaneous task.

For Study 2 we predicted that children's spelling performance would be inconsistent across the tasks. At the text-level, spelling processes need to be integrated with composition processes in a spontaneous spelling task, but not in the other one, dictation. Thus, we proposed that children’s spelling performance would change as a function of task, depending on how automatic the spelling process is.

## Study 1: Cross-Task Consistency between Dictation and Spontaneous Spelling at the Word-Level

In Study 1, 1^st^ graders’ performances in a standardized spelling test (dictation) were compared to their performances in a spontaneous spelling measure at the word level of language.

### Method

#### Participants

A total of 132 Italian children participated in this study (Age: 5.86±.38; 67 boys and 65 girls). Participants were 1^st^ graders located in the same school district in a mid-sized city in Central Italy, characterized by a medium-high socio-economic level with Italian as their first language. Participants’ parents gave written informed consent for their children’s participation. Data was collected at the beginning of the school year. In the Italian educational system, children typically start kindergarten when they are three years old and finish when they are five. Children then start primary school when they are six years old. Primary school lasts five grades. The school year begins in mid-September and ends in mid-June. In Italy, formal literacy teaching begins in primary school, and follows a specific curriculum, as set down in national law. All participating schools were following the national guidelines provided by the Ministry of Education and no participating school was following an alternate program.

All examiners were psychologists, trained in research and methodology in educational psychology. They received specific training on how to administer each of the tests included in this study. The authors of this paper did not participate either in the examination or in the scoring phase. All tests were collected in schools, during school hours. Principals and teachers previously agreed with the aims and procedures of this study. The measures were administered at a time agreed upon with the school and with due adherence to the requirements of privacy and informed consent required by the Italian law (Law Decree DL-196/2003). Regarding the ethical standards for research, the study referred to the last version of the Declaration of Helsinki [50]. This study was approved by the Ethics Committee of the Department of Psychology at the University of Florence, Italy.

#### Measures

Children's spelling performance was assessed through an Italian language standardized test [51], which includes a dictation of words task and a dictation of pseudo-words task, and through a spontaneous spelling task.

***Spontaneous spelling*:** Participants were asked to write as many words as they could in 10 minutes. The correctness score for this task was calculated based on the number of errors (phonological and non-phonological) committed during spelling, balanced for the total number of syllables. Agreement between judges was 97% and disagreements were resolved through discussion.

***Dictation spelling*:** This measure was assessed through a standardized test [51]. It includes two sub-tests, word dictation, and pseudo-words. Participants had to write down 18 bi- or tri-syllabic high-frequency words (e.g., mattina [*morning*] and padre [*father*]) and 9 bi- or tri-syllabic pseudo-words (e.g., fosto and gnoba) dictated by the experimenter. The two correctness scores (word and pseudo-words) were calculated based on the number of errors (phonological and non-phonological) committed during dictation, balanced for the total number of written syllables. Agreement between judges was 98% and disagreements were resolved through discussion. The reliability scores were good, i.e., α coefficient = .88 for words, and .89 for pseudo-words.

#### Data analysis

The principal descriptive statistics (mean, standard deviation, skewness and kurtosis coefficients) were carried out. Variables were non-normally distributed and the applied monotonic transformation did not produce any effect. Thus, we tested the research hypothesis through non-parametric statistical tests. To test the correlation between spontaneous spelling and dictation we calculated a Spearman's Rho correlation score for the total group. To determine whether participants committed more orthographic errors in the dictation tasks (words vs. pseudo-words) or in the spontaneous spelling task, we performed two Wilcoxon signed-rank tests. Finally, as a post-hoc test to better interpret the results of the Wilcoxon test, we dichotomized the dictation and spontaneous spelling test inaccuracy scores into two groups, that is good spellers (scores lower than the 85^th^ percentile), and poor spellers (scores higher than the 85^th^ percentile). This cut-off score was chosen to identify both students with a spelling disability (5^th^ percentile, [47]) and students with a spelling difficulty. A Fisher’s exact test was calculated to compare the frequency of good spellers and poor spellers in the three standardized spelling tests (spontaneous spelling, dictation of words, and dictation of pseudo-words), and to verify whether frequency distributions were independent or dependent across tasks. The Fisher’s exact test was used instead of the chi-square because of the small sample size and because not all expected frequencies in cells were greater than 5. We calculated odds ratio as a measure of effect size. All analyses were performed by spelling error.

### Results

The database for study 1 is available in S1 Dataset. In study 1, 95 (73.6%) children were good spellers across all the three tasks; 18 (14%) were poor spellers in one task only, 10 (7.8%) were poor spellers in two tasks, and 6 (4.7%) were poor spellers in the three tasks. Table 1 reports the descriptive statistics of the total sample as well as the statistics of poor vs. good spellers. For all variables, the skewness and kurtosis score were > 1, thus we tested our hypothesis through non parametric tests. The error scores correspond to the number of spelling errors made by children, whereas the syllable score corresponds to the exact number of syllables written by the child. The ratio score represented the number of errors made divided by the number of syllables written.

**Table 1:** Standardized test descriptive statistics: number of participants, mean, standard deviation, median and interquartile range.

		Poor spellers				Good spellers				Total			
Variable	Measure	N	M	Mdn	IQ	N	M	Mdn	IQ	N	M	Mdn	IQ
Spontaneous spell.	Errors	19	10.16±5.89	9	8	111	3.51±3.82	2	4	130	4.48±4.78	3.00	5.25
	Syllables	19	62.32±25.87	60	35	111	106.67±66.35	88	86	130	100.50±64.84	81.00	71
	Ratio	19	.16±.08	.14	.07	111	.03±.03	.03	.03	130	.05±.06	.03	.06
Word dict.	Errors	19	35.89±17.41	35	20	113	8.71±6.38	9	10	132	12.62±12.97	10.50	12.00
	Syllables	19	35.42±12.41	44	19	113	38.44±9.16	44	16	132	38.01±9.69	44.00	16.00
	Ratio	19	1.04±.38	1	.32	113	.22±.15	.20	.23	132	.34±.35	.25	.31
Pseudo-words dict.	Errors	20	19.50±10.91	16.5	15	111	5.09±3.51	5	4	132	7.29±7.38	6.00	6.00
	Syllables	20	18.05±6.92	20	10	111	21.86±3.68	24	8	132	21.12±4.86	24.00	8.00
	Ratio	20	1.11±.44	1.03	.61	111	.24±.16	.21	.21	132	.37±.39	.25	.33

*Note*. IQ = Interquartile range

Spontaneous spelling correlated with both dictation task sub-tests: words (r = .20, p = .02) and pseudo-words (r = .37, p < .001). The two dictation test sub-tasks showed a high correlation (r = .78, p < .001).

#### Difference in spelling performance across tasks

Children’s spelling was poorer in word dictation (*Mdn* = .25) than for spontaneous spelling of words (*Mdn* = .03) (*Z* = -9.17, *p* < .001, *r* = .57). Children’s spelling was poorer in pseudo-word dictation (*Mdn* = .25) than for spontaneous spelling of words (*Mdn* = .03) (*Z* = -9.49, *p* < .001, *r* = .59). Both comparisons reported large effect sizes (*r* > .50).

#### Cross-task independency for spelling performances at the word level

To determine whether performances were independent or dependent across tasks, a Fisher’s Exact test was performed. Performance in spontaneous spelling of words and word dictation were dependent on each other, *p* < .05, odds ratio = 8.25. Performance in spontaneous spelling of words and pseudo-word dictation were also dependent on each other, *p* < .01, odds ratio = 8.17.

### Discussion

In this study, we examined the cross-task independency between spelling in dictation and spelling in spontaneous spelling at the language level of words. The fact that spontaneous spelling and dictation only moderately correlated confirms Berninger and Whitaker's suggestion [52] to include both spelling assessments in school, as they are probably supported by different sub-processes; pseudo-word dictation task activates the sub-lexical route.

The comparison of students’ spelling performances across tasks confirms that dictation represents a more difficult task than spontaneous spelling. Typically, teachers and experimenters adopt dictation to assess spelling, as it allows them to include difficult words; the ones that confuse children. Results also showed that students’ performances in the dictation and spontaneous spelling tasks are dependent on each other. Although considered to be a transparent orthography, Italian also presents a few exceptions. For instance, words with the graphemes QU/CU/CQU or SCE/SCIE-CE/CIE, GE/GIE that may be spelt in different ways and require lexical procedure to be spelt correctly, without the necessity of referring to the sentence context [53]. At the word-level children are unable to use the context (*i*.*e*., the sentence or the text) to determine which is the correct spelling, and have to rely only on their sub-lexical and lexical processing. Although dictation is more difficult than spontaneous spelling, this effect is systematic for all children, which suggests that word-spelling performances are quite consistent across tasks (i.e., dictation vs. spontaneous spelling), confirming previous research on preschoolers [2]. Regarding the word choice, it is likely that children spontaneously wrote words they already knew as orthographic patterns, retrieving them directly from their semantic storage, unlike the dictation task in which they did not have orthographic patterns for 1/3 of the words (the pseudo-words). Even so, prior study suggests that at the beginning of first grade, most of the children rely on the sub-lexical route only [5,42]. Thus, it is likely that students used this strategy for both tasks.

## Study 2: Cross-Task Consistency between Dictation and Spontaneous Spelling on the Text-Level

In Study 2, 1^st^ graders’ performances in a standardized spelling test (dictation) were compared to their performances in a spontaneous spelling measure (narrative writing) at the text level of language.

### Method

#### Participants

81 Italian children participated in this study (Age: 6.8±.35; 44 boys and 37 girls). The participants attended grade 1 in primary school located in the same school district in a mid-sized town in Central Italy, characterized by a medium-high socio-economic level. Participants’ parents gave informed consent for their children’s participation. All participants spoke Italian as their first language. Data was collected at the end of the school year.

#### Measures

Children's spelling performance when writing a text was assessed through two tasks, a spontaneous spelling task—created by the researchers—and a dictation task–derived from a standardized test available for the Italian population [54].

***Spontaneous spelling*:** Children wrote a narrative to their liking in a maximum of 60 minutes and were completed in between 30 and 50 minutes. The inaccuracy score was calculated based on the number of errors (phonological and non-phonological) committed, balanced for the total number of words written. Agreement between judges was 95% and disagreements were resolved through discussion. The reliability score was good.

***Narrative quality [55]:*** The quality of children's narratives was assessed through two macro-level components, i.e., structure and coherence (see prior studies by the authors for more details on the measures, *e*.*g*., [24,26]). Structure was coded on a 5-level scale on the basis of presence/absence of eight fundamental story elements: (a) title, (b) conventionalized narrative opening, (c) characters and setting, (d) problem, (e) central event, (f) resolution, and (g) conventionalized narrative closing. These *five levels were*:

1^st^ level (no narrative): simple description or list of events, objects, or facts;
2^nd^ level (sketch narrative): opening, setting, character(s), conclusion or opening, sketch of the problem, and resolution;
3^rd^ level (incomplete narrative): opening, character(s), problem, and resolution;
4^th^ level (essential narrative): opening, character(s), problem, central event, and resolution;
5^th^ level (complete narrative): title, opening, character(s), setting, problem, central event, resolution, and narrative closing.

Coherence was coded on a 3-level scale on the basis of the number of incoherencies, balanced for the total number of propositions. An example of incoherence is a sentence introduced by an adversative even though it did not contradict the previous sentence. Based on the number of incoherencies per total number of propositions, we assigned the narratives to four categories of coherence: absent; low (the ratio of incoherencies/propositions was below the 33^rd^ percentile); medium (the ratio of incoherencies/propositions was between the 33^rd^ and 66^th^ percentiles); and high (the ratio of incoherencies/propositions was above the 66^th^ percentile).

***Dictation spelling*:** This measure was assessed through a standardized test [54]. Children wrote down a 59-word dictation ('La bicicletta di papà', Eng. tr. 'Dad's bicycle'). The inaccuracy score was calculated based on the number of errors (phonological and non-phonological) committed during dictation, balanced for the total number of written words. Agreement between judges was 96% and disagreements were resolved through discussion. The reliability score was good, i.e., α coefficient = .87.

#### Data analysis

To test the hypothesis of study 2, we ran the same set of analyses as study 1 (see section of data analysis for study 1). All analyses were performed by spelling error. Additionally, in this study we compared the narrative qualities in terms of structure and coherence through a series of Students’ t tests for independent samples, a test particularly indicated for mean comparisons between groups with different sample sizes.

### Results

The database for study 2 is available in S2 Dataset. In study 2, 60 (75%) children were good spellers across all the three tasks; 16 (20%) children were poor spellers in one task only; and 4 (5%) children were poor spellers in both tasks. Table 2 reports the descriptive statistics of the total sample as well as the statistics of poor vs. good spellers. For all variables, the skewness and kurtosis score were > 1, thus we tested our hypothesis through non parametric tests. The error scores correspond to the number of spelling errors made by children, whereas the syllable score corresponds to the exact number of syllables written by the child. The ratio score represented the number of errors made divided by the number of syllables written.

**Table 2:** Standardized test descriptive statistics: number of participants, mean, standard deviation, median and interquartile range.

		Poor spellers				Good spellers				Total			
Variable	Measure	N	M	Mdn	IQ	N	M	Mdn	IQ	N	M	Mdn	IQ
Dictation	Errors	12	23.05±5.91	20	10	69	5.59±4.38	5	7	81	10.33±9.17	7.00	14.00
	Syllables	12	53.36±7.21	55.5	5	69	57.85±.52	58	0	81	56.63±4.23	58.00	1.00
	Ratio	12	.46±.24	.36	.20	69	.10±.08	.09	.12	81	.20±.21	.12	.25
Narrative	Errors	12	21.58±12.41	17.5	9	68	5.09±6.79	3	5	80	7.56±9.78	3.50	8.00
	Syllables	12	57.33±30.89	48	29	68	50.49±32.66	40	30	80	51.51±32.30	41.00	30.00
	Ratio	12	.38±.13	.35	.04	68	.09±.07	.07	.09	80	.13±.14	.09	.13
	Structure	12	3±.95	3	2	68	2.85±1.06	3	2	80	2.90±1.06	3	2
	Coherence	12	1.75±.97	1	2	68	2.47±.86	3	1	80	2.36±.90	3	2

*Note*. IQ = Interquartile range

Overall, children's inaccuracy in the dictation test correlated with their inaccuracy in the narrative test (*r* = .33, *p* = .00).

#### Difference in spelling performance across tasks

First, to determine whether participants made more orthographic errors in the dictation task or in the spontaneous spelling task, we performed a Wilcoxon signed-rank test. No significant differences in spelling accuracy were found between the two tasks at the text level of language (*Z* = -1.93, *p* = .054, *r* = .15). Unlike the word-level of language, at the text level of language children need to perform well at both transcription and composition. Probably, children are more concentrated on the compositional aspect of their written narrative and tend to dedicate less cognitive resources to the transcription stage. Since the transcription process is still not fully mastered or automatic, children tend to commit mistakes in this task too. Generally, research on writing has focused on the interference effect that spelling plays on text quality [4], whereas this study suggests that in early spelling acquisition stages the reverse effect could happen where composition would interfere with spelling performance.

#### Cross-task independency for spelling performances at the text level

A Fisher Exact test was used to determine whether performance was similar across tasks. Dictation inaccuracy was independent from spontaneous spelling inaccuracy, *p* = .075.

#### Quality of children's narratives

Children's performances in structure did not differ across the groups. Good spellers in both tasks (M = 2.46, SD = .86) wrote more coherent narratives (t = 3.01, df = 59, p = .00, *d* = 1.25) than poor spellers in both tasks did (M = 1.43, SD = .79). Good spellers in the spontaneous spelling task, but poor spellers in the dictation task (M = 2.50, SD = .86) wrote more coherent narratives (t = -2.78, df = 19, p = .01) than poor spellers in both tasks did (M = 1.43, SD = .79, *d* = 1.30). Both comparisons reported large effect sizes (*d*>.80).

### Discussion

In this study we examined the cross-task independency between spelling in dictation and spelling in spontaneous spelling at the language level of texts. Similar to the word level of language, the correlation effect size was moderate, confirming Berninger and Whitaker's concerns [52] about adopting only one assessment type. The Wilcoxon test confirmed that neither task is systematically more difficult than the other. Fisher’s exact test confirmed the independency between spelling performances in the two tasks also for good and poor spellers. This inconsistency at the text level of language is explained by the fact that when writing a text, children need to integrate transcription and composition processes [46]. Thus, some children might be more concentrated on the compositional aspect of their written narrative and tend to dedicate less cognitive resources to the transcription stage. If the spelling process is not fully mastered and automatic, children tend to make mistakes also in this task. The comparison of performances in text quality between groups identified by their spelling performances suggests that if spelling skills have been automatized, the child can dedicate more cognitive resources to the construction of a mental model of the narrative, which in turn leads to writing a more coherent narrative. One the one hand, results indicate that children who can spell have better narrative writing ability. One the other hand, the reverse may also be so, that children who have a better narrative writing ability can also spell better. The current research design does not allow us to provide a clear direction of the link between spelling and narrative. Past studies brought some evidence to the first hypothesis, as spelling in first grade was found to be a mediator of the relationship between oral narrative competence in kindergarten and written narrative competence in first and second grade [24].

## General Discussion

This contribution aims at increasing our understanding of children’s early acquisition of spelling. Specifically, we explored the dependency of first graders’ spelling performances in a dictation versus a composition task, and examined whether the relationship between dictation and spontaneous spelling varies at the language level of word and text. On a theoretical level, both these variables should introduce different processes. In a dictation task, the child needs to transcode words that are externally dictated and thus limit the possibilities for internally constructing the words, whereas in a composition task the child can choose which words to transcode. However, at the language level of text, in a dictation task the child needs to simply transcode, and cognitive resources can be fully dedicated to this process, whereas in a composition task the child needs to integrate the transcoding with other cognitive processes (e.g., planning, revising, monitoring, and the like) and cognitive resources need to be balanced between these two.

The results of the two studies suggest the existence of differences in the relationship between spelling to dictation and spelling to compose from the word to the text level of language. Whereas at the word-level the spelling performances in the dictation and composition tasks are dependent on each other, at the text-level they are only moderately correlated, but the spelling performance in a task did not predict the spelling performance in the other task within subjects. One way to explore the reciprocal relationship between spelling and text quality is to analyze poor spellers’ narratives and compare them to good spellers. The fact that children with a good mental model of the narrative (as assessed by coherence) were also able to write more correctly, brings evidence to the hypothesis that spelling and composition are strictly interrelated processes [24], and that focusing more on one does not necessarily mean that less cognitive resources are available for the other. Even when the spelling process is not fully mastered yet, and children fail in the dictation task, they are still able to write coherent and correct stories.

Results can also be interpreted in light of the characteristics of Italian, a language with a transparent orthography. In most languages spelling is more difficult than reading, but this difficulty gap is enhanced in transparent orthographies, in which the regularity of the orthographic system is higher in grapheme-phoneme relations (forward regularity) than it is in phoneme-grapheme relations (backward regularity) [5,56]. At the word level of language, there are only a few exceptions, which include words with the graphemes QU/CU/CQU or SCE/SCIE-CE/CIE, GE/GIE [53]. Instead, at the text level of language, the ambiguities in the phoneme-grapheme conversion drastically increase. For instance, *anno* (year) and *hanno* (they have) are homophone words, but children have to rely on the context to decide which spelling is correct. Thus, at the word level, spelling performance in a dictation or spontaneous spelling task might be dependent on each other, since the children can correctly spell all the items through the sub-lexical route, which is well developed already at early stages of spelling acquisition. This is different from what happens with deep orthographies, in which children have to immediately rely on the lexical route, also to spell words. Instead, at the text level of language, children might need to rely on the lexical route to disambiguate the spelling of homophone words, similarly to what happens in deep orthographies.

Overall, the results from these two studies indicate that the relationship between the dictation and the spontaneous spelling task changes at different levels of language (word and text). The text triggers a different way of representing words. Indeed, when children spontaneously spell words (study 1) they seem to activate the same processes as they do when words are dictated, whereas when children spontaneously spell a text, individual differences play a fundamental role. In particular, we isolated the effect of students’ narrative competence, and demonstrated that spelling processes are strictly anchored to it when children are writing a text. Instead, when children are passively spelling a text that someone is dictating to them, they seem to consider it as a list of words, rather than a system of meanings. This effect might depend on the modalities of dictating. Indeed, often teachers dictate a text much slower than the average speed for narrating it, with long pauses between one word and another. Future studies should confirm this hypothesis by testing children’s spelling performances in two tasks, spontaneous composition and dictation, by varying the speed through which the text is dictated.

A few limitations affected these two studies. Firstly, the present research design does not allow one to directly compare word and text language levels. Future studies could have language level as a within-subject variable. In this way, results could have the potential to show whether spelling words out of context and spelling words in text are different processes or not. Secondly, our understanding of cross-task consistency of spelling competence is limited to the word- and text-level. Future studies should also include other levels of language, e.g., the sentence-level. Indeed, at this level, children have to focus predominantly on syntactic aspects of grammar, which might affect their spelling competence differently. Although the syntactic skills are involved at the text-level too, it is integrated with other high-level skills, making it difficult to isolate this specific level of language.

The findings of this research have relevant practical implications, as they help not to misinterpret spelling problems in both direction and underestimating or overestimating a spelling impairment [2,5,7]. Current practices of spelling assessment have received several criticisms. Berninger and Whitaker [52] argued that both spelling procedures should be adopted as they moderately correlate and their variance is explained by different process measures. Few authors [6,57] critically questioned the use of standardized tests for the assessment of spelling, in particular because there is a mismatch between what these tests ask one to do and normal day to day spelling experiences. More ecological measures, for example authentic assessment or curriculum-based measures, that focus on composition assessment have been proposed as ways of devising tests that are better matched with everyday experiences.

## Supporting Information

S1 Dataset Database in cvs format for Study 1. (CSV)

S2 Dataset Database in cvs format for Study 2. (CSV)
